# Rescaling scientific communication

**DOI:** 10.1098/rsnr.2016.0034

**Published:** 2016-08-24

**Authors:** Mark Patterson

**Affiliations:** eLife Sciences Publications, Ltd, 1st Floor, 24 Hills Road, Cambridge CB2 1JP, UK

Like the revolution in scientific communication that took place in the nineteenth century, today's information revolution is driven by new technology and a sharp decrease in the costs of the distribution of scientific knowledge. Digital media and networked communications, the analogues of cheap printing and better transport infrastructure in the nineteenth century, have unleashed tremendous potential for improved and more effective communication.

One of the most striking differences, however, is that the current transformation is taking place against the backdrop of an existing large and commercially highly profitable publishing industry. Coupled with an incentive system in science that places heavy emphasis on publication in established journals, the incumbent publishing system has tended to maintain the status quo at the expense of new approaches.

Set against these brakes on innovation are some drivers for change, some of which find parallels with the situation two centuries ago. For example, the struggle that scientists have with the volume of information is not new. In the modern day this information comprises a vast peer-reviewed journal literature, where not only are there more articles to read but there is also a tendency for articles to contain an ever-increasing amount of data. New channels of communication dedicated to different kinds of research output have emerged to deal with the growth in the numbers of researchers and the scale and variety of the products of their work.

More effective methods for sharing and using research findings mean that new audiences can be connected with scholarship, and an entirely different and very potent type of ‘reader’ is the computer itself. Computational tools are being developed that will assist, for example, with the information overload problem by helping scientists and others to navigate and interrogate the literature. Through text mining approaches, scholars could be guided towards findings that are of most interest to them. The effectiveness of such approaches is tightly linked with the extent to which the literature is freely available without limitation on reuse—the vision of open access and an important part of the revolution that is now underway.

Perhaps the central challenge that needs to be addressed, however, in the context of research communication is the incentive system in science. The belief that a successful career depends on publication in specific journals, rather than on the quality of the work that is conducted, has led to a level of competition to publish in these journals that many now feel is unhealthy to the scientific enterprise, for example, by undermining the reliability and reproducibility of science.

One way to tackle this concern is to reform practices of research assessment. Instead of focusing on the names of the journals in which work is published, evaluation processes should focus on the specific scientific contributions that have been made. Such reform could also place greater emphasis on core scientific values such as openness, objectivity, cooperation and collaboration.

The reinvention of scientific incentives is largely a cultural challenge, but new technology can help. There are now tools to monitor the online reception and usage of a new scientific article. The evidence of influence can be both quantitative (such as numbers of citations or social bookmarks) and qualitative (such as how a study has influenced policy or practice). Importantly, these approaches can also be applied to research outputs other than research publications, including data, code, protocols and other resources.

As demonstrated by the inexorable increase in the extent of open access literature across all of scholarship, the adoption of new and better approaches for research evaluation will increase. Rather than the narrow focus on traditional research articles as the currency of scholarship, we should see greater attention paid to other forms of research output, possibly coupled with a (welcome) reduction in the volume of traditional articles.

In the nineteenth century the number of science journals increased from about 100 to about 10 000 journals. Perhaps one aspect of the digital revolution in scholarship will be a reversal in the earlier trend—from 10 000 to 100 ‘journals’. A smaller number of very large and open channels of communication, each specializing in a different type of output or content might be a very effective foundation upon which new tools and services are provided to support the work of research communities and the scientific enterprise as a whole.

